# GMP-compliant ^68^Ga radiolabelling in a conventional small-scale radiopharmacy: a feasible approach for routine clinical use

**DOI:** 10.1186/s13550-015-0105-3

**Published:** 2015-04-24

**Authors:** Roeland Vis, Jules Lavalaye, Ewoudt MW van de Garde

**Affiliations:** Department of Clinical Pharmacy, St Antonius Hospital, Koekoekslaan 1, 3430EM Nieuwegein, The Netherlands; Department of Nuclear Medicine, St Antonius Hospital, Koekoekslaan 1, 3430EM Nieuwegein, The Netherlands

**Keywords:** ^68^Ga PET imaging, NET, DOTA-NOC, Octreotide, GMP, Validation

## Abstract

**Background:**

The number of routine care patient examinations with ^68^Ga radiopharmaceuticals is still relatively limited, probably caused by the presumed need for large investments in hot cells, automated synthesis modules, laboratory equipment and validation efforts. Our aim was to set up the preparation of ^68^Ga-DOTA-NOC in compliance with all current European Union-Good Manufacturing Practices (EU-GMP), current Good Radiopharmacy Practice (cGRPP) and European Pharmacopoeia (Ph. Eur.) guidance but without the availability of a hot cell and gas chromatography (GC), high-performance liquid chromatography (HPLC) and atomic absorption spectrometry (AAS) equipment.

**Methods:**

A risk-based approach was applied to align preparation conditions with applicable regulations, together with a validation of a thin-layer chromatography (ITLC) method to replace HPLC as modality for examining radiochemical purity.

**Results:**

Using an internally shielded labelling module for manual operation, a ^68^Ga-DOTA-NOC labelling procedure was set up that meets all applicable Ph. Eur. specifications. The applied ITLC method showed very good correlation with HPLC results (*r* = 0.961) and was able to detect relevant deviations in radiolabelling procedures. All identified quality assurance aspects were made compliant with EU-GMP and cGRPP guidance.

**Conclusions:**

We consider the described configuration and validation approach feasible for many conventional small-scale radiopharmacies, something that could help to increase the availability of ^68^Ga radiopharmaceuticals to a large number of patients.

## Background

Within the field of nuclear medicine, there has been an interest in the clinical applications of the ^68^Ge/^68^Ga generator for several decades. Already in 1960, Gleason described the ‘positron cow’, which at that time was used for the compounding of ^68^Ga-EDTA for the imaging of brain tumours [1]. With a physical half life of 68 min, availability independent of a cyclotron and excellent binding characteristics to chelators such as DOTA ^68^Ga has excellent properties for routine clinical application. The most widely implemented application of ^68^Ga at present is the imaging of somatostatin receptor positive tumours. This application, most specifically in the imaging of neuro-endocrine tumours (NET), has taken off with the development of ^68^Ga-DOTA-TOC, ^68^Ga-DOTA-NOC and ^68^Ga-DOTA-TATE, all analogues of octreotide with differing affinity for the somatostatin receptor subtypes 2, 3 and 5 [2-5]. The 2010 European Association of Nuclear Medicine (EANM) guidelines for PET/CT tumour imaging with ^68^Ga-DOTA-conjugated peptides states that positron emission tomography (PET) with ^68^Ga-DOTA-TOC, ^68^Ga-DOTA-NOC and ^68^Ga-DOTA-TATE has brought about dramatic improvements in spatial resolution in the imaging of NET as compared to conventional imaging with SPECT tracers [6].

After several decades of merely being promising, it seems that ^68^Ga radiopharmaceuticals are finally ‘breaking through’. The number of publications concerning ^68^Ga radiopharmaceuticals has increased drastically during the past years: the number of ^68^Ga-related scientific articles published in 2011 to 2012 accounts for over 45% of all publications since 1956 [7]. Furthermore, several recent publications of the highly promising new tracer, ^68^Ga-labelled Glu-urea-Lys(Ahx)-HBED-CC ([^68^Ga]Ga-PSMA-HBED-CC), for the imaging of prostate cancer have come available, thereby significantly expanding the clinical possibilities of ^68^Ga radiopharmaceuticals within oncology outside of the relatively small number of patients with NET [8,9].

Despite the recent advances, the availability of ^68^Ga radiopharmaceuticals to patients is still relatively limited. An estimated 10,000 scans are being performed yearly in Europe at about 100 centres using ^68^Ga-labelled somatostatin analogues; for the USA, only 2 university sites have begun to undertake human studies [10]. In the Netherlands, ^68^Ga radiopharmaceuticals for routine clinical use are currently available in three hospitals only, two of which are university medical centres. Several factors for this past-hampered clinical introduction of ^68^Ga radiopharmaceuticals can be identified and include a scarcity of ^68^Ga generators, absence of ^68^Ga radiopharmaceuticals with a marketing authorisation (absence of radiopharmaceutical cold kits) and lack of PET radiopharmaceutical regulations [7]. Presently, several Good Manufacturing Practices (GMP)-grade generators are commercially available, as well as different (semi-)automated synthesis modules [7,11]. In 2014, the first generator received a marketing authorisation in the European Union (EU). Moreover, with the publication of the European Pharmacopoeia (Ph. Eur.) monographs on ^68^Ga-chloride and ^68^Ga-edotreotide (DOTA-TOC) injection and the EANM guideline on the small-scale ‘in-house’ preparation of radiopharmaceuticals which are not kit procedures (cGRPP) and the existing EU-GMP annexes’ very specific guidance and quality requirements are currently provided [12-15]. Although these developments have reduced the number of barriers to implement ^68^Ga radiolabelling for routine clinical use, several still remain. The major barriers involve the required investment in hot cells, a synthesis module and quality control equipment (e.g. gas chromatography (GC), high-performance liquid chromatography (HPLC) and atomic absorption spectrometry (AAS) suitable for and dedicated to radiopharmaceuticals) and validation efforts. These prerequisites are more easily met in well-equipped (often university hospital) radiopharmacy departments but less often in conventional small-scale radiopharmacies. This situation could hamper the widespread utilization of ^68^Ga-radiopharmaceuticals in the future, thereby denying large numbers of patients access to state-of-the-art nuclear medicine diagnostics. Our aim was to set up the preparation of ^68^Ga-DOTA-NOC in compliance with all current EU-GMP, cGRPP and Ph. Eur. guidance but without the availability of a hot cell and GC, HPLC and AAS equipment.

## Methods

### Facilities and equipment

**Figure 1:**
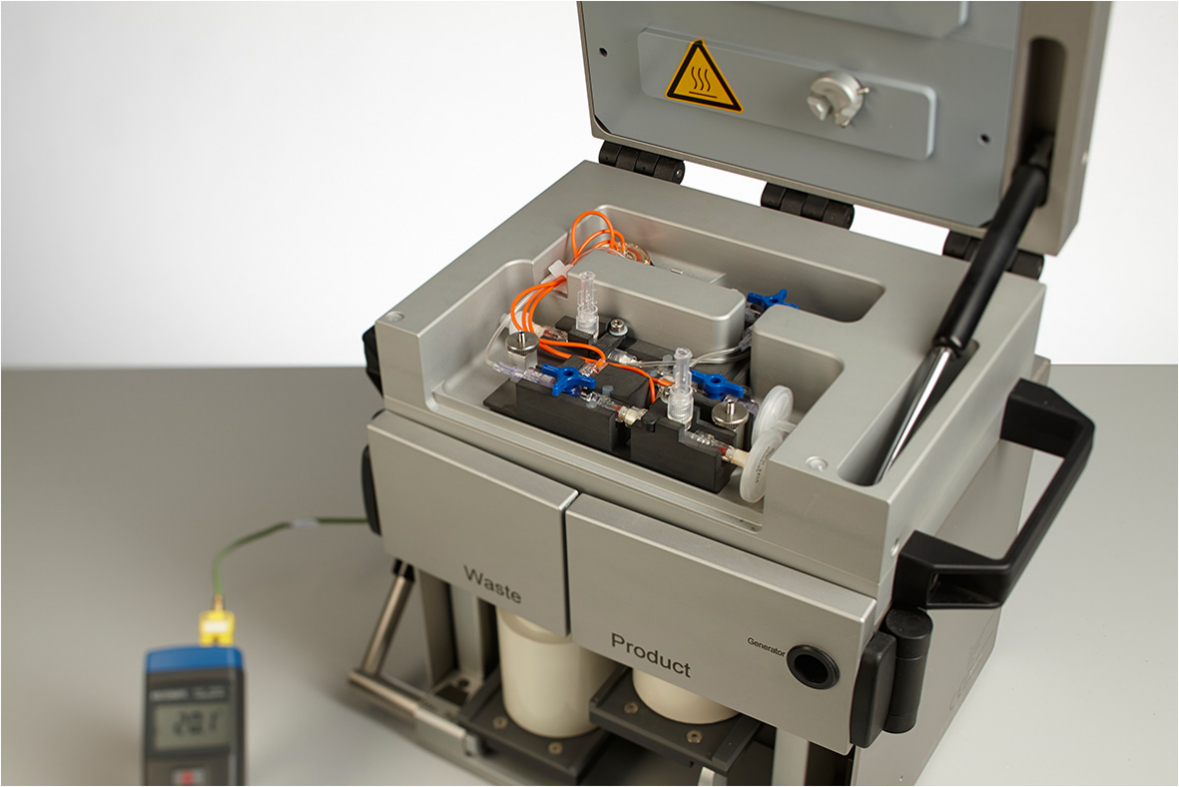
The labelling module.

The radiopharmacy department of the St Antonius Hospital (850-bed teaching hospital), Nieuwegein, The Netherlands, situates two cleanrooms, one with GMP grade D and one with GMP grade C air quality. Within the latter cleanroom, one GMP grade A workstation (lead-shielded unidirectional laminar airflow workbench (LAFW)) is placed. For labelling of ^68^Ga-radiopharmaceuticals, a labelling module for manual operation and a GMP grade 925 MBq ^68^Ge/^68^Ga generator were obtained from ITG (Gärching/Munich, Germany). The labelling module (Figure 1) is internally lead-shielded and situated on a standard workbench in the background environment. Placing the labelling module in a GMP grade C background is in accordance with the Annex 3 GMP and cGRPP guideline for on-line sterile filtration in a closed system under the condition that the aseptic assembly of all components down-stream of the filter is performed in a GMP grade A environment [14,15].

The ITG labelling module holds an internal block heater wherein a disposable reaction vessel (part of the disposable cassette) is placed. The ^68^Ge/^68^Ga generator is situated in the bottom section of the module, and a disposable cassette system (per batch) is placed in the top section. After installation of the cassette system, the (lead-shielded) upper lid of the module is closed, thereby providing lead shielding of both the reactor vessel and all tubing. All solutions needed during the labelling can be introduced by means of plastic syringes through small openings in the upper lid or in front of the module. Because the labelling module is designed for manual non-electrical operation, software validation according to the GAMP-5 methodology was not applicable [16]. Furthermore, no specific cleaning validation was applicable because all materials that might contact the product are for single use only.

All radioactivity measurements are performed with a standard ionization chamber (VIK-202; Comecer, Joure, The Netherlands).

### Preparation and process controls

For radiolabelling, the ITG GMP grade ^68^Ga radiolabelling kit and single-use sterile cassette were used (ABX Advanced Biochemical Compounds, Radeberg, Germany). GMP-grade DOTA-NOC acetate in 60 μg vials was obtained from ABX (Radeberg, Germany). ABX was approved as a supplier in conformance with our quality management system based on their GMP manufacturing authorisation and a supplier audit.

#### Generator elution and general labelling procedure

Prior to every labelling, the generator is rinsed with 0.05 M hydrochloric acid unless the generator has been eluted within the previous 24 h. Dependent on the period from last elution (24 to 72 or >72 h) the rinsing volume is 10 or 20 ml, respectively. The rinsing solution is transferred to a waste vial directly. For every labelling procedure, a sterile single-use cassette is installed in the labelling module. The C18 purification cartridge is conditioned with 2 ml 70% (*V*/*V*) ethanol, purged with 1 ml air and subsequently rinsed with 5 ml sodium chloride (NaCl) 0.9%. The assembly of the sterile final product vial with needle and 0.2 μm product filter and venting needle/filter is performed under aseptic conditions in the LAFW (grade A environment) and transferred to the grade C environment for assembly to the cassette. The connection between the cassette and the final product vial is upstream of the sterilizing filter.

For the labelling procedure, 60 μg DOTA-NOC acetate was dissolved in 150 μl of 0.25 M sodium acetate buffer. After switching on the block heater within the labelling module at a fixed time, a volume of 100 μl DOTA-NOC (40 μg) is added with a calibrated microlitre pipette (range 10 to 100 μl) to a volume of 1 ml 0.25 M sodium acetate buffer and subsequently injected in the reaction vessel. After a heating time of 5 min, direct elution of the ^68^Ge/^68^Ga generator is performed with 4 ml 0.05 M hydrochloric acid into the reaction vessel. Labelling takes place at 90°C to 95°C for 10 min. Subsequently, the labelling mixture is transferred to the waste vial through the C18 purification column and rinsed with 5 ml 0.9% NaCl. In the final step, the C18 purification column is eluted with 1 ml 60% (*V*/*V*) ethanol and rinsed with 5 ml 0.9% NaCl through the on-line 0.2 μm filter into the product vial.

#### Routine in process controls

Labelling is performed according to a paper worksheet by two operators, and all process steps are signed off by both operators. After installation of the cassette, a leak test is performed with 0.9% NaCl to verify that no leakage occurs. Temperature settings are signed off as well as the temperature readings at set time intervals during the labelling. The percentage of waste activity is documented for information purposes only, although the measure can be indicative of a labelling failure (unbound ^68^Ga).

### Quality control of the ^68^Ge/^68^Ga generator

Considering the non-metallic matrix of the ITG ^68^Ge/^68^Ga generator, a validation study was performed to assess the necessity for routine testing for iron and zinc. We therefore took eluate samples after (1) 1 week after last elution of the generator, (2) 1 day after last elution of the generator and (3) 3 h after last elution of the generator. The iron and zinc contents were determined by means of inductively coupled plasma (ICP) by the Department Radiation, Science and Technology (RST), section Radiation and Isotopes in Health (RIH) at the Delft University of Technology, the Netherlands.

For the eluate from the generator, there is no requirement of sterility, but there is a requirement for bacterial endotoxins of less than 175 IU/V with *V* the maximum volume to be used for the preparation of a single patient dose [12]. Since we elute the ^68^Ge/^68^Ga generator in the grade C environment and use non-sterile hydrochloric acid, we performed a validation study to evaluate the introduction of bioburden and endotoxins due to non-aseptic generator elution conditions. For worst-case purposes, we used 0.05 M hydrochloric acid that was of ultrapure, laboratory grade that was not tested for bioburden or endotoxins. Eluate samples for bioburden and bacterial endotoxins were obtained (1) after leaving the generator stagnant for 1 month and (2) 2 h after rinsing with 30 ml hydrochloric acid. The endotoxin content was determined by means of a kinetic LAL assay (Kinetic-QCL®, Lonza, Walkersville, MD, USA).

Another quality parameter for ^68^Ge/^68^Ga generators is the breakthrough of the long-lived parent radionuclide ^68^Ge (impurity A in the monograph for ^68^Ga chloride and impurity C in the monograph for ^68^Ga-edotreotide) and its content in the final radiopharmaceutical preparation. Therefore, upon receipt of the ^68^Ge/^68^Ga generator, we performed a ^68^Ge breakthrough test of both eluate and labelled ^68^Ga-DOTA-NOC according to the Ph. Eur.

Based on the acquired results, an overall evaluation was made to assess the applicability and control methods of all parameters defined in the Ph. Eur. monograph on ^68^Ga-chloride.

### Quality control of ^68^Ga-DOTA-NOC

Since no HPLC apparatus is available for radiopharmaceuticals at the St Antonius Hospital, initial verification of the labelling procedure was performed at ITG and involved the preparation of three validation batches in a time course of 2 days. Quality control was performed with HPLC in accordance with the Ph. Eur. monograph directly after labelling and for one batch after a maximum of 5.5 h of storage at ambient temperature in either a glass vial or a polypropylene syringe [13]. Parallel with one batch, a cross-validation study was performed to verify the suitability of two different thin-layer chromatography (ITLC) methods for unbound ^68^Ga (impurity B) to replace the HPLC method from the Ph. Eur. monograph. Fresh ^68^Ga-DOTA-NOC was ‘spiked’ with impurity B (fresh eluate) to simulate labelling efficiencies in the range of 85% to 100%. All batches were analyzed with three methods to determine the activity of ^68^Ga-DOTA-NOC as percentage of total activity: (1) HPLC, (2) ITLC method 1 and (3) ITLC method 2. For ITLC method 1, Merck Millipore TLC silica gel 60 F254 aluminium sheets (Billerica, MA, USA) as stationary phase, 0.1 M citric acid buffer at pH 4 to 5 as mobile phase with radiodetection after cutting the sheets in half and for ITLC method 2, Varian ITLC-SG as stationary phase, 0.1 M citric acid buffer at pH 4 to 5 as mobile phase with radiodetection after cutting the sheets in half were used. Spearman’s test for correlations was applied to HPLC *versus* ITLC method 1 and HPLC *versus* ITLC method 2 using IBM SPSS 22.0 Statistics (Statistical Package for the Social Sciences; IBM, Armonk, New York, NY, USA).

After the initial cross-validation study at ITG, the correct performance of the ITLC method for impurity B was confirmed on-site at the St Antonius Hospital. At St Antonius, radiochromatogram scanning was performed with a VCS-203 chromatography scanner (Comecer, Joure, the Netherlands). The ITLC method for impurity A was performed in conformance with the applicable Ph. Eur. monograph at all times.

Besides the test for the potential introduction of bioburden and endotoxins from the generator, a validation study was performed to assess the total bioburden and possible introduction of bacterial endotoxins from non-sterile DOTA-NOC acetate, the ^68^Ga radiolabelling kit and C18 purification cartridge. To do so, three full mock labellings were performed excluding heating to prevent the risk of bioburden reduction through heating at 90°C to 95°C. The endotoxin content was determined as described before.

The filter integrity after the final sterile filtration was assessed by conducting a filter integrity test using a Merck Millipore filter tester (SLTEST000, Merck Millipore Co., Billerica, MA, USA). After rinsing the filter with 10 ml water for injections, the bubble point is determined with a requirement of a minimum of 3,450 mbar/50 psi according to the Merck Millipore requirements. In more detail, a closed system is constructed with a sterile 50 ml Luer-lock syringe connected to the Merck Millipore filter tester, which is again connected to the filter unit. From the filter unit, a needle is placed in a 100-ml vial containing water for injections in which the bubble point is determined. This method is based on the evaluation provided by Hayashi et al. [17].

To assess the overall appropriateness of the aseptic process, three media fills were performed by direct inoculation of tryptic soy broth medium (TSB, bioTRADING Benelux B.V., Mijdrecht, the Netherlands). The aseptic assembly of all components down-stream of the filter was performed in the GMP grade A environment and subsequently TSB medium that has been air-exposed to the grade C environment was filtered. The filtered TSB medium was subsequently incubated at 30°C for 14 days.

Based on the results obtained, an overall evaluation was made to assess the applicability and control methods of all parameters defined in the Ph. Eur. monograph on ^68^Ga-edotreotide. The final labelling procedure was subsequently tested through the preparation of three validation batches on three separate days with quality control directly after labelling and for one batch after storage in a plastic syringe for 1.5 h as well.

### Radiation safety

All radiopharmacy technicians were trained in radiation protection and wear personal thermoluminescence dosimeters (TLDs) (NRG, Petten, the Netherlands) which are evaluated monthly. To assess the body dose during a labelling, a study was performed with measurement of total dose during rinsing of the ^68^Ge/^68^Ga generator and radiolabelling procedure at three locations including the body of the radiopharmacy technician. For this study electronic personal dosimeters were used (ThermoFisher Scientific Inc., Waltham, MA, USA).

## Results and discussion

### Quality control of the ^68^Ge/^68^Ga generator

The assay for iron and zinc in the generator eluate samples after (1) 1 week after last elution of the generator, (2) 1 day after last elution of the generator and (3) 3 h after last elution of the generator resulted in levels of iron of 1.8, 3.7 and 0.91 μg/GBq respectively and levels of zinc of 10, 1.2 and 1.8 μg/GBq respectively. This means that the results were in conformance with the Ph. Eur. requirement of a maximum of 10 μg/GBq for all samples although the zinc level in the eluate after resting of the generator for 1 week was at the Ph. Eur. maximum. These findings are in accordance with the decay scheme of ^68^Ga yielding zinc but also confirm that the non-metallic matrix of the generator in itself is no source of iron or zinc. Considering this, the introduction of a standard rinsing procedure of the generator prior to eluting for labelling seems sufficient and will prevent the need for AAS equipment for routine testing of iron and zinc.

After the ^68^Ge/^68^Ga generator had been left stagnant for 1 month, we observed a bioburden of 300 colony forming units (cfu)/ml in the eluate, which was reduced to <10 cfu/ml (no growth) after the standard rinsing procedure. The endotoxin content of both elutions were <0.250 IU/ml and therefore much lower than the requirements of 175 IU/V ml. The bioburden and endotoxin levels of the 0.05 M hydrochloric acid are controlled and specified on the certificate of analysis. We consider the verification of this certificate sufficient and therefore do not perform a routine assay for bioburden or bacterial endotoxins on the ^68^Ga eluate.

The ^68^Ge breakthrough test of the eluate showed a breakthrough of 0.0009% which is below the limit from the Ph. Eur monograph of ≤0.001%. The ^68^Ge breakthrough in the final product was equal to zero in the batch tested and confirms the effectiveness of the post-labelling purification step by C18 cartridge. In our opinion, the only clinically relevant content of ^68^Ge is in the final product and not in the eluate. Also considering that the procedure for assessment of ^68^Ge breakthrough in the final product is not feasible in routine practice (measurement after full decay of ^68^Ga), a ^68^Ge breakthrough test at acceptance of every new generator could be sufficient.

In summary, the validation work on the quality control of the ^68^Ge/^68^Ga generator in our specific configuration showed that based on a risk-based approach and the results from the validation studies, the tests on iron, zinc, ^68^Ge-breakthrough and endotoxins can be limited to an initial qualification of the generator only (see Table 1).

**Table 1:** **Selected parameters from the Ph. Eur. monograph on** ^**68**^ **Ga-chloride and how they are controlled**

**Parameter**	**Specification**	**Method**
Iron	Maximum 10 μg/GBq	Quality by design: risk-based approach eliminated need for routine test
Zinc	Maximum 10 μg/GBq	Quality by design: risk-based approach eliminated need for routine test
Bacterial endotoxins	Less than 175 IU/V to be used for the preparation of a single patient dose	Quality by design: risk-based approach eliminated need for routine test
Impurity A	≤0.001%	Ionization chamber: performed at time of acceptance of generator

### Quality control of ^68^Ga-DOTA-NOC

**Figure 2:**
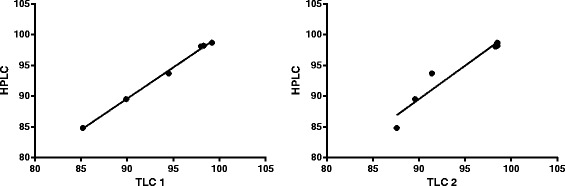
Correlation between HPLC and the two ITLC methods.

The results of the HPLC *versus* ITLC validation are summarized in Table 2. The cross-validation work for impurity B showed good correlation between HPLC and the two ITLC methods (ITLC method 1: *r* = 0.999 and ITLC method 2: *r* = 0.961, see Figure 2). Although the correlation between HPLC and ITLC method 1 shows somewhat better, we consider method 2 more appropriate given the much shorter run time for the mobile phase over the stationary phase. This shortens the time needed for quality control between preparation and administration to the patient. Based on the validation results obtained, we concluded that ITLC method 2 can be considered appropriate to eliminate the need for HPLC to assess impurity B from the Ph. Eur. monograph.

**Table 2:** **Results of validation of HPLC** ***versus*** **ITLC (presented as 100% minus impurity B)**

**Sample**	**Percentage of Ga-68-DOTA-NOC (HPLC)**	**Percentage of Ga-68-DOTA-NOC (ITLC 1)**	**Percentage of Ga-68-DOTA-NOC (ITLC 2)**
Validation 1	98.2	98.3	98.5
Validation 2	98.1	98.0	98.3
Validation 3	98.7	99.2	98.5
Spike 5%	93.7	94.5	91.4
Spike 10%	89.5	89.9	89.6
Spike 15%	84.8	85.2	87.6

Another test from the Ph. Eur. monograph for ^68^Ga-edotreotide injection is a HPLC method for a test on edotreotide (maximum 50 μg per maximum dose) [13]. Because in our labelling procedure only 40 μg DOTA-NOC is used, this test can be considered not applicable. The correct amount is assured by applying vials with a maximum content of 60 μg and verifying the correct reconstitution and drawing of the required amount by two radiopharmacy technicians. These steps are recorded on to the paper worksheet.

Also the assessment of total ethanol content and impurity D (HEPES) in the final dose ^68^Ga-DOTA-NOC appeared not applicable after a risk evaluation. The total amount of ethanol introduced in the final product is 0.47 g or 10% (*V*/*V*) which is far below the absolute maximum of 2.5 g per administration and conform the requirement of 10% (*V*/*V*). This calculation prevents the need for routine GC analysis for ethanol content. The ITLC test on impurity D is not required because no HEPES buffer is used in the preparation process.

For all mock labellings, we observed a bioburden of <10 cfu/ml (no growth) and endotoxin content of <0.125 IU/ml. This endotoxin content is far below the threshold of 175 IU/V ml (see before). When considering these results together with the specifications of the sterilizing filter used, we conclude that the pre filter bioburden is far below the maximum capacity of the filter. The Cathivex® GV filter (Millipore) used for on-line sterile filtration employs the Millipore Durapore® 0.22-μm membrane which is validated to withstand a *challenge* with 4.0 × 10^7^ of the micro-organism *Brevundimonas diminuta*. These numbers are even much higher than the worst-case bioburden of 300 cfu/ml observed after leaving the ^68^Ge/^68^Ga generator stagnant for 1 month (see before). Based on this validation work and evaluation, we consider that a routine analysis of bioburden or bacterial endotoxins is not warranted except for a successful filter integrity test as a release criterium. This approach is also supported by the successful validation of the aseptic process showing no bacterial growth after incubation of the media in any of the three simulation experiments. According to the GMP principles, this aseptic validation will need to be repeated biannually together with a personal aseptic qualification of all technicians.

In summary, some tests in the Ph. Eur. monograph on ^68^Ga-edotreotide can pose a problem in the absence of GC and HPLC equipment. The validation work on the quality control of ^68^Ga-DOTA-NOC in our specific configuration showed that based on a risk-based approach and the results from validation studies, the tests on quantity of DOTA-NOC, ethanol content, endotoxin levels and impurity D need not be performed on a routine basis. The test for impurity B can be replaced by an ITLC method. An overview of all controlled parameters, specifications and test methods considered applicable to the labelling procedure presented is provided in Table 3. Table 4 shows the results from the three validation batches prepared in our facility prior to the release of the final labelling procedure for routine clinical use. Finally, the configuration and validation approach described above does apply to ^68^Ga-DOTA-NOC, and many aspects may require additional validation efforts when labelling other peptides. Furthermore, any configuration should always comply with the local quality management system.

**Table 3:** **Selected parameters from the Ph. Eur. monograph on** ^**68**^ **Ga-edotreotide and how they are controlled**

**Parameter**	**Specification**	**Method**
Identity DOTA-NOC	DOTA-NOC	Acceptance of raw material
Content: ^68^Ga	90 – 110% of the declared ^68^Ga radioactivity	Ionization chamber^a^
Content: DOTA-NOC	Maximum 50 μg	Quality by design: pre-filled vials with amount needed together with double check in batch record^a^
Appearance	Clear, colourless solution	Visual inspection^a^
pH	4.0 to 8.0	pH indicator strip^a^
Ethanol	Maximum 10% (*V*/*V*)/maximum 2.5 g per dose	Quality by design: risk-based approach eliminated need for routine test
Sterility	Sterile; bubble point >3,450 mbar	Validation of aseptic process together with filter integrity test^a^
Bacterial endotoxins	Less than 175 IU per dose	Quality by design: risk-based approach eliminated need for routine test
Impurity A	≤3%	ITLC^a^
Impurity B	≤2%	Validated ITLC method to replace HPLC^a^
Impurity C	≤0.001%	Ionization chamber; assessed at time of acceptance of generator

^a^Parameter with corresponding method that is performed after every preparation of ^68^Ga-DOTA-NOC.

**Table 4:** **Results of validation batches (directly after labelling)**

**Parameter**	**Validation batches (** ***n*** **= 3)**
Appearance	Clear, colourless solution
Mean pH (range)	5.0 (5.0)
Filter integrity	Pass
Mean impurity A (%) ± SD	0.94 ± 0.54
Mean impurity B (%) ± SD	0.21 ± 0.18

SD, standard deviation.

### Radiation safety

Personal dosimetry with routine TLD monitoring did not reveal an increase in radiation exposure to the body.

The total personal radioactivity dose during rinsing of the ^68^Ge/^68^Ga generator with 30 ml 0.05 M hydrochloric acid (originally required after >72 h of no elution) was found to be 2 μSv. The personal radioactivity dose during the subsequent ^68^Ga radiolabelling procedure observed was 1 μSv. This implies that one rinsing of the generator followed by a labelling procedure corresponds to 3 μSv. Unfortunately, we did not assess the finger dose during drawing of the final product into a syringe and during administration to the patient. For the specific purpose of evaluation of possible labelling strategies, however, this can be considered as less relevant because drawing into a syringe and administration to a patient will be required anyhow independent of the labelling strategy applied.

## Conclusions

The described implementation and validation approach for the preparation of ^68^Ga-DOTA-NOC is shown to result in a robust labelling strategy that complies with all current EU-GMP, cGRPP and Ph. Eur. guidelines and monographs, but without the need for a hot cell, radiochemical HPLC, GC or AAS equipment. It is our strong belief that with this type of validation approach, the implementation of ^68^Ga radiolabelling should be feasible in much more nuclear medicine facilities than apparent today. Adopting these practices could aid in increasing the availability of ^68^Ga radiopharmaceuticals to a large number of patients.
